# Tailored machine learning models for functional RNA detection in genome-wide screens

**DOI:** 10.1093/nargab/lqad072

**Published:** 2023-08-21

**Authors:** Christopher Klapproth, Siegfried Zötzsche, Felix Kühnl, Jörg Fallmann, Peter F Stadler, Sven Findeiß

**Affiliations:** Leipzig University, Department of Computer Science and Interdisciplinary Center of Bioinformatics, Bioinformatics Group, Härtelstrasse 16-18, D-04107 Leipzig, Germany; ScaDS.AI Leipzig (Center for Scalable Data Analytics and Artificial Intelligence), Humboldtstraße 25, D-04105 Leipzig, Germany; Leipzig University, Department of Computer Science and Interdisciplinary Center of Bioinformatics, Bioinformatics Group, Härtelstrasse 16-18, D-04107 Leipzig, Germany; Leipzig University, Department of Computer Science and Interdisciplinary Center of Bioinformatics, Bioinformatics Group, Härtelstrasse 16-18, D-04107 Leipzig, Germany; Leipzig University, Department of Computer Science and Interdisciplinary Center of Bioinformatics, Bioinformatics Group, Härtelstrasse 16-18, D-04107 Leipzig, Germany; Leipzig University, Department of Computer Science and Interdisciplinary Center of Bioinformatics, Bioinformatics Group, Härtelstrasse 16-18, D-04107 Leipzig, Germany; Max Planck Institute for Mathematics in the Science, Inselstraße 22, D-04103 Leipzig, Germany; University of Vienna, Institute for Theoretical Chemistry, Währingerstraße 17, A-1090 Vienna, Austria; Santa Fe Institute, 1399 Hyde Park Rd., Santa Fe NM 97501, USA; Universidad Nacional de Colombia, Facultad de Ciencias, Bogotá, D.C., Colombia; Leipzig University, Department of Computer Science and Interdisciplinary Center of Bioinformatics, Bioinformatics Group, Härtelstrasse 16-18, D-04107 Leipzig, Germany

## Abstract

The *in silico* prediction of non-coding and protein-coding genetic loci has received considerable attention in comparative genomics aiming in particular at the identification of properties of nucleotide sequences that are informative of their biological role in the cell. We present here a software framework for the alignment-based training, evaluation and application of machine learning models with user-defined parameters. Instead of focusing on the one-size-fits-all approach of pervasive *in silico* annotation pipelines, we offer a framework for the structured generation and evaluation of models based on arbitrary features and input data, focusing on stable and explainable results. Furthermore, we showcase the usage of our software package in a full-genome screen of *Drosophila melanogaster* and evaluate our results against the well-known but much less flexible program RNAz.

## INTRODUCTION

Experimental and theoretical work in molecular biology typically presupposes the correct annotation of genomic data and therefore depends on the compilation and curation of accessible sequence databases. This demand for high-quality genomes led to the emergence of high-throughput sequencing (HTS) techniques and huge amounts of data since the 2000s (1,2).

Automated annotation pipelines increasingly supplement – and to a certain extent replace – time consuming, error-prone and often poorly reproducible manual curation techniques (3). Still, until recently, annotation efforts have largely focused on protein-coding genes (reviewed by (4)). However, less than 25% of the transcribed human genes account for the entirety of the 19 000 protein-coding RNAs (5). Non-coding RNAs (ncRNAs) have been shown to perform a diverse range of biological and regulatory functions (6,7) and play an important role in the genesis of cancer and other pathophysiological processes (8). They are much more diverse than protein-coding RNAs with respect to their biogenesis, processing, molecular mechanism, and evolutionary histories. While some prominent classes, such as microRNAs, siRNAs, snoRNAs, rRNAs and tRNAs (9) are well understood, information is still sparse for most ncRNAs. These molecules display a wide range of features such as folding and assembling into complex superstructures, interactions with other RNAs, DNA or proteins, and the regulation of their activity (10). Non-coding RNAs have also been found to play a major role in chromatin remodeling and epigenetics (11). Despite their abundance and biological relevance, identification and annotation of ncRNAs is still much sparser and more superficial than the functional annotation of protein-coding genes. At least in part this is explained by a preference for poly-A enrichment protocols in HTS-based studies, and by the predominant interest in protein-coding genes in medical research (12). As a consequence, protein-coding genes have also received more attention in computational studies.

**Figure 1.**
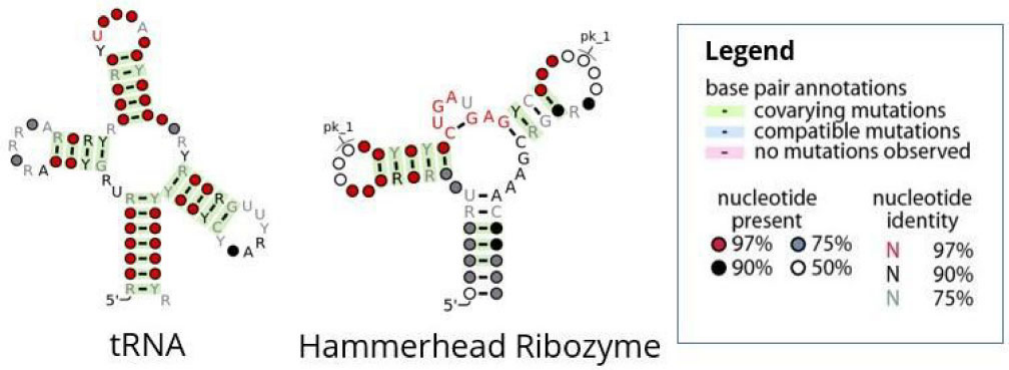
Secondary structure is a common indicator for biological function in the annotation of non-coding RNAs. Many examples, such as the depicted tRNA (left) and Hammerhead Ribozyme (right), show a particularly high degree of conservation of a common structure and a strong evolutionary selection towards mutations that stabilize it. The high degree of observed co-varying and compatible mutations strongly support the hypothesis that their structure is required for the sequence to retain its structure-to-function relationship. Figures were generated using R2R (13).

A subset of well-described non-coding RNAs features a significant degree of conservation of their secondary structures, which is often associated with biological function (14,15), Figure 1. Evolutionary conservation of RNA secondary structure therefore serves as a promising predictor of function in the *de novo* identification of ncRNA elements. Both minimum free energy (MFE) structures and intrinsic properties of nucleotide sequences are used in tools such as RNAz (16,17) or Evofold (17) to identify evolutionary conserved, and thus likely biologically functional, secondary structures. Such approaches are facilitated by the relative ease of predicting RNA secondary structures for a given sequence using a simple, well-understood energy model (18). Recent studies suggest that the sequence of a given RNA motif can evolve at normal or even accelerated pace while conserving the associated secondary structure, thus suggesting evolutionary pressure targeted specifically at conservation of structure configuration (19). There is also evidence for a high diversity of functional motifs, such as structural switches and others, that is not yet fully mapped out even in bacterial regulatory pathways (20).

The majority of recently developed automatic annotation pipelines focus on the classification of confirmed transcripts, e.g. from high-throughput RNA sequencing experiments, as non-coding RNAs or, more common, long non-coding RNAs (lncRNAs) against a background of protein-coding sequences. Prime examples of such tools include CPAT, CPC2 and CNCI (21–23). Effectively, these methods define ncRNAs as everything that is not recognized as protein-coding, instead of assuming an ambiguous background. The limitations of such a two-way classification approach for the identification of long non-coding RNAs were already discussed elsewhere (24). In the context of a screen conducted on genomic alignment data, it has to be noted that large parts of the genome are only transcribed in specific cell types (25,26).

While the reality of pervasive transcription of genomic DNA is widely accepted, it has remained a matter of debate what fraction of the transcriptional output conveys recognizable biological function and whether there are high levels of ‘transcriptional noise’ (27). The latter view is supported in particular by the low levels of sequence conservation observed for most lncRNAs, although the majority of transcriptional units are evolutionarily old (28). Classification of transcribed sequences, furthermore, is only viable if the available transcriptome data is sufficiently accurate to assemble essentially complete full-length transcripts. This is often not the case due to comparably low expression levels. Taken together, modeling an ambiguous background is of utmost importance when screening sequences of unknown function, as is the case with genome-wide screens for identification of potentially transcribed loci of interest and their hypothetical function.

Machine learning methods are utilized in many automatic annotation pipelines in the field of comparative genomics (29,30). A key issue, however, remains the inability of trained models to adapt to other species not sufficiently represented in the training data. This poses a serious problem since features like nucleotide frequencies, codon usage, and structure motifs are distributed unequally across different realms of life. Therefore, one-size-fits-all approaches typically cannot tap their full potential as they have to forego properties of high discriminative power specific only to a smaller set of related species.

To address this issue, we implemented Svhip (Software and manual accessible under: https://github.com/chrisBioInf/svhip), a novel software pipeline for the calibration of diverse machine learning models used in genome-wide non-coding and/or protein-coding RNA screens. The software is available as source code on Github as well as provided via the conda environment manager. The key feature of Svhip is the ability to train a multitude of different models automatically while giving full control over individual steps to the user. We offer the possibility to generate both models for the identification of structurally conserved non-coding RNA elements against an ambiguous background as well as for the differentiation between non-coding RNAs, protein-coding sequences and/or undefined others. Generated models can be used in the framework of Svhip itself or, in the case of two-class models, be exported to be used with the highly successful annotation tool RNAz (16,31). Long-term, we aim towards creating a diverse collection of models for various applications working out of the box, as well as enabling users to share their own models and parameter sets. This, by itself, ensures a large amount of reproducibility and accountability to individual experiments and genome screens and their resulting discoveries.

The Results section of this contribution focuses on new developments and the evaluation of automatically trained Svhip classifiers, while the Materials and Methods section summarizes in detail how data has been acquired and processed. In the latter we first summarize how test data has been compiled that is used to evaluate prediction accuracy of existing approaches, i.e. RNAz and RNAcode, and the models generated with Svhip. Second, the processing of raw alignment data for model training is described. This includes random background model generation and a detailed description of well established and newly developed features. The Materials and Methods section is complemented by the description on what kinds of models Svhip supports, how they are trained and how their quality is accessed. Finally, data acquisition for an exemplary genome wide screen on *D. melanogaster* is summarized. All this is utilized to present Svhip’s applicability to classify non-coding RNA elements and/or protein-coding sequences against an ambiguous background throughout the Results section.

## MATERIALS AND METHODS

### Test Data preparation

Two separate data sets were used in the evaluation process of Svhip. For initial testing, we used a preexisting data set that was originally applied to evaluate RNAz (31) and SISSIz (32). It consists of 3,832 established and structurally conserved alignments with 2–6 sequences of non-coding RNAs sourced from the Rfam database (33,34). A wide range of vertebrate species is covered in this set. As a control, it also contains a set of random genomic locations taken from full genome alignments, which mirrors the non-coding set in length, number of sequences and dinucleotide composition.

To test the capabilities of our own genomic background simulation approach for future data set generation, we shuffled each non-coding RNA alignment on a column by column basis using the rnazRandomizeAln.pl tool from the RNAz software framework. We also simulated alignments based on the non-coding set with conserved dinucleotide composition and gap patterns utilizing SISSIz. Therefore, the data set consists of four subsets (non-coding, random genomic locations, column shuffled and SISSIz-simulated) with 3,832 alignments each. This data set was later reduced in size to 3,060 alignments in each subset, as all alignments containing only two sequences have been removed. This was necessary, because SISSIz shows some substantial drawbacks in the simulation of such alignments, see Supplementary Figure S1. If not otherwise indicated, this is the data set used in evaluations.

A second data set is based on a 27-way full genome alignment with the model organism *Drosophila melanogaster* as reference provided by the UCSC genome browser (35,36) (https://hgdownload.soe.ucsc.edu/goldenPath/dm6/multiz27way/maf/). The corresponding annotation in GTF format was obtained from FlyBase (version 6.44, last accessed on March 20, 2022) (37,38). To prepare full genome data for later assessment and comparison with the RNAz software, alignments were sliced into overlapping windows with the rnazWindow.pl software on a per-chromosome basis. We used windows of length 120 nucleotides and a step length of 40 nucleotides, generating an expected overlap of 80 nucleotides between neighboring windows. This rather large overlap is necessary to reduce the likelihood of falsely excluding genetic loci that display only very localized conservation signals. It should be noted that the parameters listed here are not mandatory for Svhip, but are considered optimal for most RNAz use cases. As we want to achieve a direct comparison with RNAz on a per alignment basis, we therefore followed its original protocol as closely as possible.

True labels of *Drosophila* alignment windows for evaluation purposes were assigned using the annotation in GTF format as follows: For each window we calculated the overlap with annotated transcripts, either coding or non-coding, as a fraction of the total window length. If the overlap exceeded 0.5, we assigned the corresponding label to the window. The cutoff at 0.5 was chosen to leave room for disadvantageous window slices and to increase the likelihood of at least one window covering most genes with sufficient length for detection. The alignment windows that were not filtered are then considered as representative of their respective class and serve as the basis for the generated training set. We use five features to capture essential information from these alignments while keeping redundancy at a minimum.

### Data processing

The data processing pipeline is explicitly intended for the preparation of raw alignment data for model training. It is not to be used for prediction runs, as the filtering steps implemented here may disrupt signals. Svhip accepts multiple sequence alignments in *Clustal* or *MAF* format as input. In the first processing step, these are grouped by the label assigned to them during initialization, either ncRNA or other in the case of two-way classification or ncRNA, protein-coding and other for three-way classification. We then use the rnazSelectSeqs.pl script for the initial selection of sequences. All sequences with a pairwise identity beyond a certain threshold (defaul is 98%), are rejected. This is to ensure that redundancy in the data set is reduced to a minimum.

**Figure 2.**
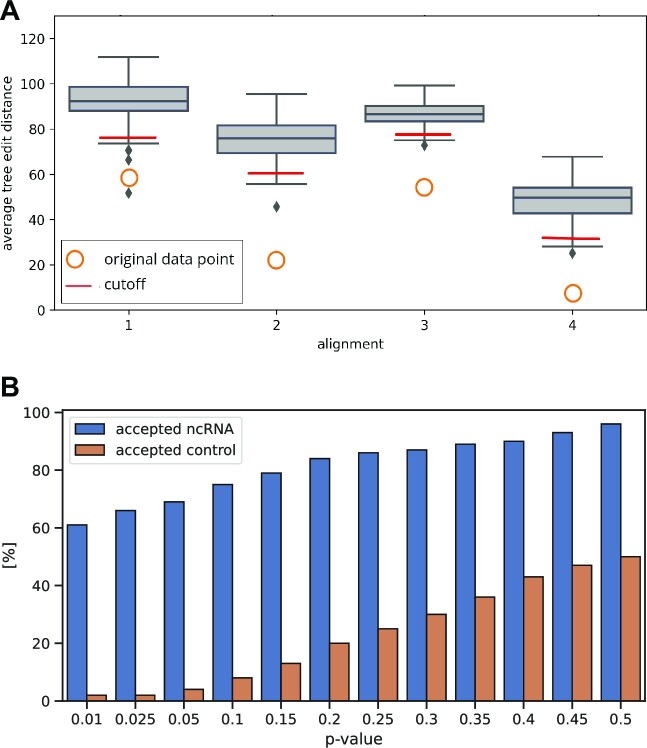
The effect of column-wise shuffling on the average tree edit distance of the sequences in an alignment. The tree edit distance between two sequences of the alignment is derived from the tree representations of the secondary structure corresponding to the minimum free energy states. (**A**) Average tree edit distances of four selected alignments (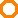) and the resulting distribution obtained by column-wise shuffling of these alignments (box-plot). Red lines indicate the estimated cutoff for statistical significance with a *P*-value of 0.05. (**B**) Possible outcomes of this filtering approach using different *p*-value cutoffs. The figure compares the acceptance rates of 100 randomly chosen strongly conserved non-coding RNA alignments and 100 alignments of random genomic locations as a control group. While it can be observed that the acceptance rate for conserved ncRNA elements remains relatively high at 60% even at a strict cutoff of 0.01, the acceptance of control alignments becomes increasingly unreliable with higher *p*-values. For this reason, a low *P*-value cut-off is usually preferable to reduce the false positive rate.

All alignments in the set are cut into overlapping windows of lengths 50–200 nucleotides. These windows then form the basis for the calculation of feature vectors for subsequent model training. Structure conservation is considered a prime indicator of functional RNA elements, windows representing the ncRNA subset of the data are filtered for this property. Alignment windows are shuffled column-wise and a tree representation of the most stable secondary structure for each sequence is calculated utilizing RNAfold. Subsequently, the average tree edit distance is calculated using RNAdistance for the window as an approximation of the remaining structure conservation after shuffling. Both of these tools are part of the ViennaRNA package (39). This step is repeated for all windows and a Gaussian background distribution is fitted to the observed average edit distances. This distribution is then used to filter the input alignments by calculating an empirical *p*-value of their average tree edit distance, which is considered significant if it is lower than 0.05, see Figure 2A. This ensures that alignment fragments with a low structure conservation do not dilute the ncRNA training set, as would be the case with, for example, lncRNAs containing long unstructured stretches. In highly conserved alignments, the column-wise shuffling ensures a reduction in structure but not sequence conservation. An acceptance statistic for 100 randomly chosen alignments from the pool of our 3,832 non-coding RNA alignments or genomic locations can be viewed in Figure 2B.

**Figure 3.**
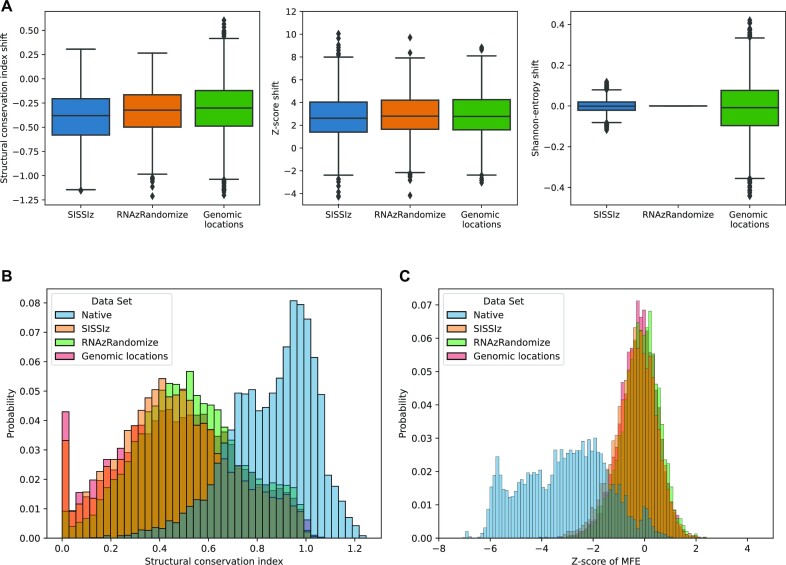
Comparison of different approaches for the generation of negative instances in the context of test set assembly. (**A**) Observed offsets (shift) relative to the input alignments using different control generation methods for the features Structural conservation index, *z*-score of MFE and Shannon-entropy. The software tools SISSIz and rnazRandomizeAln.pl are both available for the generation of training set control instances using Svhip. The Genomic locations subset is part of the original RNAz test data set and refers to genomic alignments sampled at random while attempting to preserve average dinucleotide distributions relative to the native set. (**B**) and (**C**) show the overall distributions of Structural conservation index and *z*-score of MFE, respectively, over all four data sets (one native and the three control groups).

If no negative set representing the genomic background is provided on program initialization, a synthetic negative set will be generated based on the input data. To achieve this, SISSIz is applied to simulate alignments controlled for dinucleotide composition and gap pattern. Tests show that SISSIz generated alignments can be used as a valid approximation for randomly selected genomic background yielding comparable levels of structure conservation and minimum free energy (Figure 3). It can be observed that the variance using a randomly selected genomic background is notably larger, in particular for the alignment entropy. As this value, as well as the mean pairwise sequence identity, should be as stable as possible between positive and negative sets, this also serves to illustrate the necessity of generating artificial control sets based on input data. This makes it viable to simulate an arbitrary background, while mostly preserving species-specific nucleotide distributions based on the input alignments with SISSIz. We also investigated the viability of using column-wise shuffled versions of the input alignments as negative sets (Figure 3). For this we used rnazRandomizeAln.pl, which also attempts to preserve local conservation and gap patterns during shuffling. The substantial overlap between all distributions, Figure 3B and C, indicates that all three control methods, i.e. randomly selected genomic background, SISSIz simulated and rnazRandomizeAln.pl randomized alignments, are useful and viable in principle. However, both the higher variance in sequence identity as well as the more difficult sampling process suggest to avoid the usage of random genomic locations.

The alignment windows that were not filtered are considered as representative of their respective class and serve as the basis for the generated training set. We use five features to capture essential information from these alignments while keeping redundancy at a minimum.

#### Structure conservation index

The structure conservation index (SCI) is a metric for the alignment-wide presence, size and frequency of sites that are conserved in their secondary structure. The SCI compares the MFE of individual sequences with the consensus sequence MFE. Significant deviations indicate structured elements that are not present in other sequences. Thus, a value greater or equal to 1 represents a very strong level of conservation, while a value close to 0 indicates none. The SCI for the alignment *A* is estimated by


(1) $$\begin{equation*} SCI= \frac{E_{\text{consensus}}}{\frac{1}{\vert {A}\vert } \sum _{x \in A} E_x} \end{equation*}$$

where *x* ∈ *A* are the individual sequences, *E*_*x*_ is the MFE of *x*, and *E*_consensus_ is the MFE of *A*’s consensus sequence as predicted by RNAalifold.

#### z-score of minimum free energy

The minimum free energy is a reliable indicator for the presence of paired bases and, hence, secondary structure. A point of interest in the search for conserved non-coding RNA elements is identifying (sub-)sequences with an MFE that significantly deviates from that of sequences with equal nucleotide composition but no notable secondary structure. To detect these, the *z*-score of MFE is used. It can be calculated using one of the following two procedures. In the first case, a given sequence is 100 times dinucleotide shuffled using the Altschul–Erickson algorithm (40) and the MFE is calculated for each resulting sequence. For the resulting distribution of random MFEs, the standard deviation σ and mean μ are calculated. Finally, the *z*-score of the given MFE *X* is computed as


(2) $$\begin{equation*} z = \frac{X - \mu }{\sigma }. \end{equation*}$$

**Figure 4.**
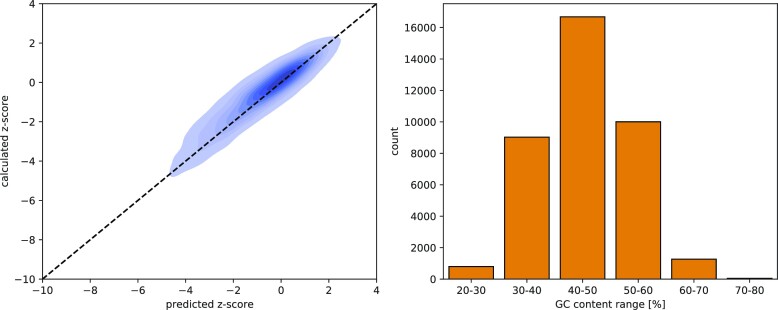
Performance of the support vector regression engine trained to predict the standard deviation and averages of minimum free energy (MFE) values of sequences with a given mono- and dinucleotide composition. A test set of 24,000 sequences was randomly selected from the non-coding and the random genomic subsets of our test data. The SVR was trained on 285,527 synthetic sequences generated to account for different GC content in 10%-intervals and different A/(A+U) and C/(C+G) ratios in 5%-steps. Left: The predicted and the calculated *z*-scores obtained from real distribution sampling. For a small number of sequences within the test set, the SVR significantly underestimates the average MFE of the shuffled sequence set (Supplementary Figure S2). The absolute mean average error (MAE) of all data points is 0.49. Right: The distribution of GC content ranges in the test set is shown. Sequences with a GC content of less than 30% and more than 70% are comparably rare in the test set. In our understanding, this accurately reflects the expected frequencies encountered in genomic test data, e.g. in the *D. mel*. genomic data analyzed as a case study.

The mean normalized *z*-score of the MFE for the full alignment can thus be expressed as:


(3) $$\begin{equation*} z_A = \frac{1}{\vert {A}\vert }\sum _{x \in A}\frac{E_x - \mu _x}{\sigma _x} \end{equation*}$$

where σ_*x*_ denotes the standard deviation, *E*_*x*_ the MFE and μ_*x*_ the mean for sequence *x*. Explicit shuffling, however, is a highly expensive operation in terms of computation time per input alignment as it effectively requires the sampling and estimation of the unimodal distribution for each sequence in the alignment. For this reason, we implemented support vector regression models that estimate the expected standard deviation and mean based on fast-to-calculate, sequence-intrinsic features. These are: all 16 dinucleotide frequencies, C to G+C nucleotide fraction, A to A+U nucleotide fraction and the normalized length of the sequence. Normalization was carried out by dividing the sequence length by the maximum considered length of 200, thereby yielding a fraction between 0 and 1. The models were trained on multiple large sets of synthetic sequences representing GC-content fractions in 0.1 intervals from 0.2 to 0.8 and sequence lengths from 50 to 200. The estimated values are within an acceptable deviation from those obtained with the exhaustive approach outlined above, see Figure 4. In our use cases, this approximation yields sufficiently accurate results. It is, however, possible to enforce a shuffling and distribution sampling for all input alignments for maximum accuracy. It should be further noted that both approaches yield reasonably stable results: The SVR will naturally return identical values for identical input vectors, the mean MFE values obtained by manual sampling in our tests typically deviate from the mean by less than 5% for 100-fold resampling (Supplementary Figure S2).

#### Shannon entropy

The Shannon entropy *H* is a metric for the sequence variation not otherwise captured by the features outlined here. It is calculated for each alignment as


(4) $$\begin{equation*} H = -\frac{1}{n}\sum _{i=1}^{n}\sum _{b\in \mathcal {B}} p_i^b \log _2 p_i^b \end{equation*}$$

where $\mathcal {B}=\lbrace A,U,G,C\rbrace$ is the alphabet of nucleobase symbols, and $p_i^b$ represents the empirical probability of nucleobase *b* at the *i*th of (in total) *n* alignment columns.

#### Alignment-wide hexamer score

The hexamer score ($\operatorname{Hex}$) is a common metric for estimating coding potential in arbitrary sequences and transcripts. A log-odds ratio is calculated based on the probability to find a given 6-mer of nucleotides in either a protein-coding or non-coding background (21,41). It is conceptually built on the observation that amino acids in a given protein influence their neighbors in their empirical likelihood to appear in this position. The score is largely dependent on the observed reading frame.

Our approach for the calculation of an alignment-wide hexamer score is as follows: first, the hexamer score is calculated for each sequence and reading frame using the formula


(5) $$\begin{equation*} \operatorname{Hex}= \frac{1}{l}\sum _{i=1}^{l}\log _2\frac{F(H_i)}{F^{\prime }(H_i)} \end{equation*}$$

where *l* is the number of possible hexamers in the sequence, *F*(*H*_*i*_) is the empirical probability to find hexamer *H*_*i*_ in a coding sequence and *F*′(*H*_*i*_) is the empirical probability assuming a non-coding background (21). As these genomic backgrounds are obviously not equal for all organisms, probability models are provided for human, mouse and drosophila genomes. However, as these are only an insufficient approximation for many research cases, the Svhip software also provides the program hexcalibrate to recalibrate these models given fasta files with coding and non-coding sequences. We then interpret the reading frame with the highest score as the ‘true’ reading frame for the purpose of evaluation, as non-coding sequences tend to produce low scores in any frame. The average value of the maximum scores is then used as the alignment-wide hexamer score. We found the hexamer score to have high predictive capabilities for the purpose of identifying protein-coding sequences. As expected it is however not suitable for the differentiation of structurally conserved regions from an unstructured background (see Figure 7).

**Figure 5.**
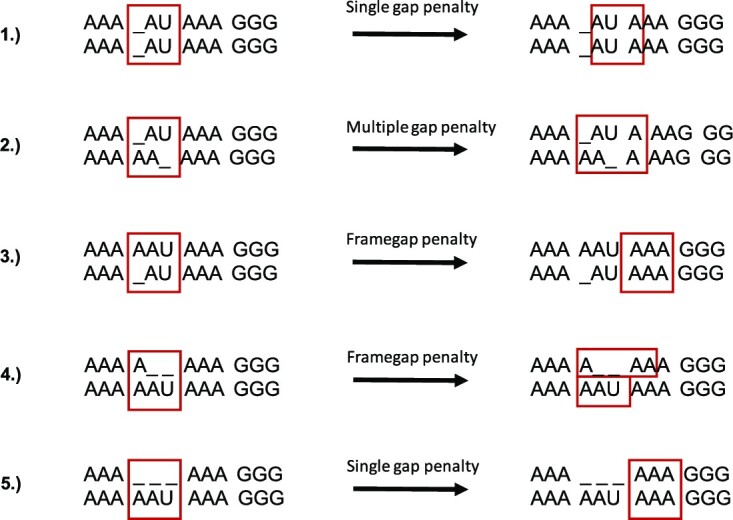
Different gap pattern handling strategies for the purpose of codon conservation calculation. The currently analyzed triplets are marked with red boxes. The left illustrates common patterns of gaps while the right hand side shows approaches how these are handled by highlighting the next analyzed triplet. 1) In the simplest case, there is just an offset by ‘aligned’ gaps that are present in both sequences. In this case, the reading frame will simply be adjusted by the number of shared gaps and a small gap penalty will be applied. 2) If gaps occur that are not ‘conserved’ between sequences but that sum up to the same number, the frame is corrected by being extended one-sided exactly by this number of gaps. 3) If there is a single gap in the non-reference sequence, it is likely that a sequence error is present here. In this case a constant penalty is applied and the gap is ignored, leaving the original (reference sequence) frame intact. 4) If there is one or a number indivisible by three gaps in the current reference sequence but not in the aligned sequence, we will assume the correctness of the reference sequence and the corresponding sequence frame is extended by that number. 5) If there are gaps that are multiples of three, the reading frame stays intact and is just shifted past the gaps. Only a slight penalty is applied to reflect the deletion of complete codons. Applied penalties are determined as follows: in case of a single or multiple gap penalty, the penalty has a value of –1 times the number of observed gaps. In case of a frame gap penalty, the value is fixed at –12.

**Figure 6.**
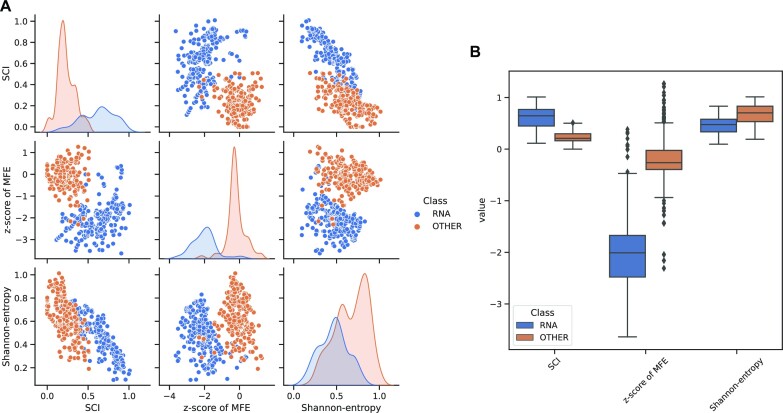
Graphical output automatically generated by Svhip, exemplified on an alignment of bacterial RNAse P RNA sequences (Rfam ID: RF0009). Every data point represents a generated alignment window incorporated into the training set (see Supplementary Table S1). For a comparison using an input file containing both eukaryotic and prokaryotic sequences as input, see Supplementary Figure S4. The graphical reports serve as a first indicator for the assessment of input data quality and separability of relevant features, thus hexamer score and codon conservation distributions are not shown for the RNA example here. (**A**) Scatter matrix showing relations between individual features as well as density plots. A good separation between native non-coding sequences and the SISSIz-generated control set can be achieved based on SCI and the *z*-score of MFE. The discrepancies between class cardinalities, as inferred from the density plots in the diagonal, can be attributed to the structural conservation filter employed in the pipeline, causing a reduction of viable data points for the non-coding RNA set. (**B**) Box plots illustrating the distribution of individual features, allowing the same observations in a more streamlined way. These reports may also serve as a basic exploratory analysis of newly assembled alignments, taking note of statistical significance of secondary structure properties as compared to the automatically generated control set.

**Figure 7.**
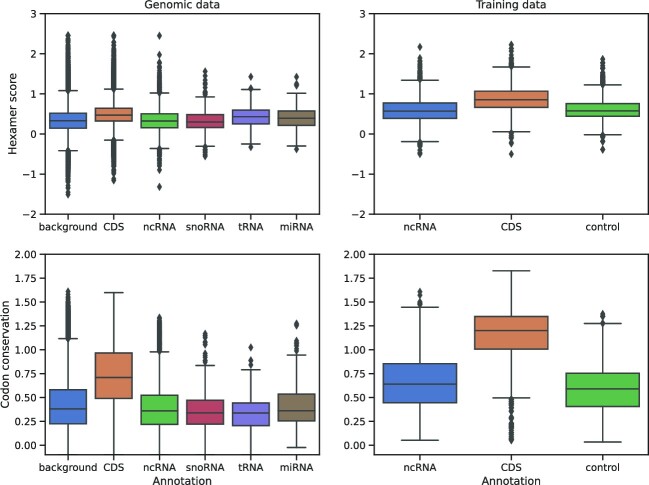
Distribution of the alignment-wide hexamer score and the codon conservation score across different gene classifications and the genomic background. Classification performance on *D. melanogaster* Multiz genome alignments (left) and applied to protein-coding alignments obtained from the InsectBase, see section Data processing, when compared with the ncRNA classification training set (right). In both cases, the codon conservation score is significantly higher in most sequences annotated as a known coding sequence. To a lesser extent, the same is true for the hexamer score.

#### Codon conservation score

In terms of alignment-based coding potential estimation, the tool RNAcode is one of the most reliable estimators, even a decade after its initial release (42). It relies on the simulation of a large number of potential phylogenetic trees to assess the statistical significance of the calculated score, which is computationally expensive. In addition RNAcode extracts the optimally scoring interval from a given alignment block and accounts for frameshifts and mis-aligned sequences. Here, we built a simplified version of the underlying scoring algorithm that sacrifices some accuracy in exchange for greatly reduced computational costs. The raw pair-wise codon conservation score (CCS) for each two sequences in an alignment is calculated as


(6) $$\begin{equation*} \operatorname{CCS}= \sum _{i=1}^{N} B62(A_i, A^{\prime }_i) - B62(A_i, A^{\prime }_i) \prod _{i=1}^3 \frac{P(t_{ij}, t^{\prime }_{ij})}{d+1} \end{equation*}$$

where $B62(A_i, A^{\prime }_i)$ is the BLOSUM62-score of the two amino acids *A*_*i*_ and $A^{\prime }_i$ encoded by the *i*th aligned codon in the first and the second sequence, respectively. *N* is the number of aligned codons. A rudimentary phylogenetic tree for the input alignment is estimated using a neighbor joining (NJ) approach based on pairwise sequence identity. The NJ algorithm is implemented using the corresponding function of the Phylo module of the Biopython library (43). This allows an estimation of the relative evolutionary distance *d* between the sequences. The default NJ method was chosen for simplicity and speed, however, custom phylogenetic trees obtained with arbitrary means are supported. $P(t_{ij}, t^{\prime }_{ij})$ is then defined as the empirical probability to observe the mutation from the nucleotide in position *j* in triplet *t*_*i*_ encoding *A*_*i*_ to the corresponding nucleotide in $t^{\prime }_i$ encoding $A^{\prime }_i$ given the tree.

The above score is calculated accounting for all possible reading frames. Errors in the alignment are skipped over by correcting for single-nucleotide deletions and frame breaks, see Figure 5. The conservation scores are stored in a matrix and used to calculate the most probable coding subregion in the alignment by calculating the maximum subarray. The score is then divided by the alignment length for normalization and used as a feature for classification.

The calculated features are saved in CSV format and can thus readily be accessed. Statistical information regarding feature distribution and degree of separability is provided as part of the output, Figure 6.

### Model training

By default, Svhip supports three different types of models: support vector machines (SVMs), random forests (RFs) and logistic regression (LR). In principle, every feature set can be used to train each type of model, even though there are some qualitative differences between them. SVMs have by far the highest computational complexity, whereas LR has the lowest (44). Furthermore, LR might not be suitable for particularly complicated classification scenarios with large overlap of feature distributions between classes, which is exemplified by the fact that many biological classification problems are not linearly separable. However, the lower training and classification time might make using an LR model worthwhile for a preliminary screen or when using an exceptionally clean data set. The question *whether SVMs or RFs* are fundamentally better in terms of raw accuracy for genomic applications is currently unanswered, even though both were capable of separating the same data sets equally well in our own studies when given appropriate training data (data not shown). When we trained RF classifiers instead of SVMs using the same training data and using the same test data, we received marginally worse accuracy results (data not shown). However, we believe that the size of the data sets is too small for generalized conclusions here.

Svhip offers automatic hyperparameter optimization for all models using either a grid search or a random walk based approach. Both options are fully customizable in their search depth and number of cross-validation steps, however, we found that more than five cross-validation iterations typically do not offer a significant improvement in accuracy given the features used (data not shown). Generated models are saved in a binary format and can be loaded using the predict option. We also note that, under certain circumstances, the expected increase in accuracy by hyperparameter optimization might be marginal when compared with the quality of the training data. Parameters associated with training, including hyperparameters and scaling parameters for normalization are saved together with the model and loaded as required for prediction purposes.

### Prediction with generated models

Svhip classifies alignment data by either employing one of the built-in prediction models, or by resorting to a custom model previously generated using the features command as described above. For the classification of input data, the alignments have to be cut into overlapping windows to achieve accurate results, analogous to their preparation for feature calculation. An alignment length of 50–200 nucleotides with a sliding overlap of 40–80 nucleotides yields reliable results for the cases presented here. For the preparation of windows, the software rnazWindow.pl of the RNAz framework was applied. The predicted class labels are assigned to the data sets on a per-window basis. Furthermore, an overall class probability is reported which, in the case of SVM classifiers, is based on a regularized maximum likelihood score (45). RFs natively support the calculation of class probabilities as a fraction of class votes by trees in the ensemble, and LR inherently calculates probabilities by virtue of being a likelihood function fitted to binary observations.

### Quality assessment of Svhip-trained classifiers

To assess the quality of the models generated by our software, we first constructed a classifier from curated Rfam data and compared the performance with the unmodified RNAz software. Sensitivity and specificity were estimated on the same data set used in the original assessment of RNAz’s accuracy (31), see section Test Data preparation. The training set was assembled by manually selecting the first 50 Rfam alignments with known and significant secondary structure as evident by the literature, see Supplementary Table S1. The alignments also had to contain more than 50 sequences. If this was the case for the seed alignment, it was used directly, otherwise we chose the regular alignment instead. These were downloaded as fasta files, and a corresponding data set was generated using Svhip with standard parameters. Based on this data, a SVM classifier was trained, using the builtin grid search hyperparameter optimization with a range of 1 to 10,000 and a logarithmic increment for the cost parameter C of the SVM. As this parameter in general governs the weight of misclassifying any given observation in a support vector machine, it is vital to optimize it for any modified training set. The same range was used as a baseline for the gamma parameter, however the auto-scaling option as provided by the scikit-learn (46) package was also added to the test range and proved to be the most efficient. The auto scaling sets the gamma value to one divided by the number of features and is, in many cases, a good estimate (46).

We note that far more care could have been taken to ensure the selection of high-quality and representative input data sets. However, an important point to demonstrate here is the underlying hypothesis that the data preprocessing pipeline as implemented in Svhip already optimizes the data set for maximum discriminative power and lowest redundancy. For this reason, we also compared the quality of the generated classifier to one using the same input data but with most of Svhip preprocessing steps deactivated. This was achieved by running the Svhipdata command with flag -F set to False and flag --max-id to 100. This deactivates both the tree-edit distance based filter for an insignificant structure signal and the filtering of (almost) identical sequences, respectively. The expectation here was a statistically significant reduction of the resulting capability to differentiate positive from negative class instances, presumably below both the optimized Svhip-generated classifier and RNAz.

### Genome-wide screen on Drosophila melanogaster

To acquire a reasonable estimate of the expected accuracy of a Svhip-trained classifier and showcase a basic experiment with a retrained classifier, we performed a genome-wide screen on *D. melanogaster* full genome Multiz alignments in MAF format and compared predicted loci with a contemporary annotation. Alignment data for *D. melanogaster* was prepared as outlined in section Test Data preparation. Two different Svhip-trained classifiers were utilized for this study: one already trained and tested in the previous section, and one with added data for protein-coding sequence differentiation.

Training data for the protein classification was assembled as follows. Coding sequences were sampled from the InsectBase (47). To this end, we randomly selected 400 protein-coding genes from the *Drosphila* annotation and then downloaded all corresponding sequences from the database. To further reduce the amount of data, we limited ourselves to the *Ephydroidea* (containing *Drosophilids*) and *Oestroidea* superfamilies. Supplementary Table S3 summarizes all protein-coding genes that were processed using the Svhip pipeline and produced feature vectors used in training.

### MAF alignment block merging

We implemented and applied mafmerge to merge short MAF blocks into larger consensus alignments. The tool, which is available as part of the Svhip software, performs the following steps: The reference genome is traversed in sense direction and for each individual alignment block, the following block is analyzed for consensus of species aligned, genomic distance and continuous reading direction between sequences aligned in both blocks. By default we use a threshold of 75% of aligned species having to be present as consensus in both blocks for merging. Furthermore, all aligned sequences have to be directly continued in the next block with respect to their genome coordinates, i.e. the start nucleotide index of one sequence has to be its end nucleotide index plus one in the other alignment block. The merging process of continuous blocks terminates automatically when a length threshold is surpassed. A default value of 1,000 was chosen based on the observation that the majority of blocks in the *Drosophila* alignments do not get joined past this point due to lack of a sufficient consenus of aligned species, see Supplementary Figure S3. Considering the compatibility of genomic coordinates of non-reference sequences implicitly adds information regarding synteny in the resulting alignments. There is no indication that this introduces biases that might adversely affect the accuracy of secondary structure prediction.

## RESULTS

We tested the Svhip pipeline and derived models for a range of different test sets and scenarios. The primary goal here was to properly estimate the specificity and expected false positive rate on both alignments of known non-coding RNAs as well as from the context of a full genome screen on *D. melanogaster*. Specifically, we also wanted to estimate the prediction quality on non-coding RNA data against the RNAz framework, which shares several of the implemented machine learning features, but comes with a hard-coded decision model, not allowing for retraining of the underlying classifier. In particular, we demonstrate that our preprocessing workflow for the raw training data does in fact yield high-quality positive instances, while the generation of synthetic negative data successfully reduces false discoveries to a minimum. We also show the separability of coding and non-coding data in an alignment context using our features designated for estimating coding potential.

### Workflow and commands

Svhip offers different modes of action, denoted as data, data3, training, features and predict. The first two serve to generate training instances for the machine learning engine from multiple sequence alignment data, either assuming a two-way (structurally conserved non-coding RNA or protein-coding RNA vs genomic background) or three-way classification (non-coding RNA, protein-coding RNA and ambiguous background), respectively. The data and data3 commands also evaluate the suitability of a given set of alignments for training the classifier by analyzing properties such as the average minimum free energy, levels of conservation and the overall quality of separation achievable with selected features. Every step of the pipeline is accompanied by graphical output to facilitate the assessment of the results and potential issues, Figure 6. The training command computes a classification model based on a data set generated with either of the previous commands. The remaining two modes, features and predict, serve to calculate the features required for prediction of alignment data, and to perform the actual classification using an existing model, respectively.

### Protein-coding features

We generalized the hexamer score, commonly used to estimate coding potential of individual sequences, to be applicable to alignments. Furthermore, a reduced version of the scoring scheme applied in RNAcode is implemented in the codon conservation score that sacrifices some accuracy for substantially improved speed, see Materials and Methods — Data processing and Supplementary Figure S5 for a comparison of run times.

As the codon conservation score was derived as a simplified version of the algorithm employed by the RNAcode software, we did a comparative analysis between both approaches on the *Drosophila* genome alignments (see section Materials and Methods — Test Data preparation). All alignment windows containing at least three sequences were screened using RNAcode and their respective codon conservation scores were calculated. The restriction of the sequence number was necessary as RNAcode does not natively support the calculation of scores for alignments of only two sequences. As RNAcode reports scores for multiple possible subalignments while the codon conservation score is alignment-wide, only the best RNAcode hit was taken into account for this comparison. The resulting Pearson and Spearman correlation coefficients of 0.49 and 0.5 indicated only a weak correlation of both scores. In part, this can be explained by the fact that the codon conservation score takes the full alignment length into account and not just the subalignment with the highest individual score. A more relevant question, however, is whether both scores are suited metrics to tell coding and non-coding sequence alignments apart. Using annotated alignment windows as a basis and separating them into *coding alignments* and *other alignments*, we performed a Wilcoxon rank sum test on both sets of data and metrics, respectively. Using this approach, the null hypotheses that both sets of measurements originate from the same underlying distribution can be rejected for both metrics with a very high confidence as indicated by *p*-values smaller than 10^−15^. Thus, while there are certain intrinsic differences in both approaches, they do achieve a strong differentiation between coding and non-coding sequence alignments.

The approach utilized by RNAcode yields higher raw discrimination power in comparison to the hexamer score (data not shown) when screening explicitly for the highest scoring, connected area in an alignment. In this sense, a protein-coding training set that was pre-screened using the RNAcode approach would, in most cases, be of higher reliability than one relying exclusively on the alignment-wide hexamer score. The former is however associated with significantly higher computational cost in an already computationally expensive pipeline. We therefore leave the decision for either of the methods to the user.

By combining information of InsectBase and FlyBase, we generated protein-coding alignments and investigated the distribution of the alignment-wide hexamer score and codon conservation score in coding and non-coding context. The codon conservation score shows a very strong separability of classes just based on this feature alone, while the alignment-wide hexamer score exhibits a moderate performance, Figure 7. The separation is much stronger in the training data set than for the genomic alignment data. This, however, is to be expected as a large number of genomic alignment windows naturally overlap with non-coding sequences, thus reducing the overall yield. The training instances can therefore be seen as an ideal classification set, depending on input data. It should also be kept in mind that both features measure different properties of coding sequences and thus naturally might produce results of varying specificity depending on the input data. In particular, the hexamer score measures the relationship of codons and their immediate neighbors, while the codon conservation score estimates the overall retention of selection for coding potential in an evolutionary context. It has to be noted that, in this classification task, no single feature carries the full information for a correct label assignment. In most cases, all of them have to be considered in the context of the remaining parameters.

### Analysis of two-way classification performance on alignments of known ncRNAs

**Figure 8.**
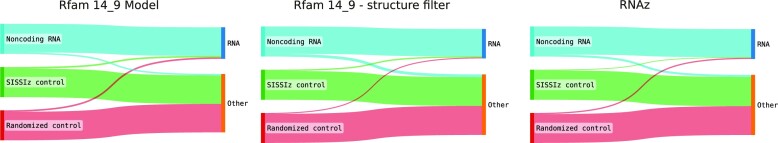
Label assignment of classifiers, comparing RNAz (right) and an optimized Svhip classifier (middle) as well as another Svhip-trained classifier using the same training set but without structure conservation significance filter (left). The test set originally contained the 3,832 alignments of known non-coding RNAs used to benchmark RNAz, which was reduced to 3,060 alignments by removing all those with only two sequences. Negative sets were generated using SISSIz and rnazWindow.pl for column-wise shuffling. Both control sets were based on the native ncRNA alignments. Removal of alignments with only two sequences was deemed necessary, as generation of a fair control set is less often possible for these (see Figure 3). The raw numbers of classifier assigned labels is summarized in Supplementary Table S2.

Accuracy, sensitivity and specificity of RNAz and two Svhip-trained classifiers were compared on the original RNAz test set. The difference between the latter two lies in their optimization by filtering for secondary structure significance of the input. The structure filtered classifier refers to the one trained by applying the secondary structure filter method and serves to illustrate the relative effectiveness in increasing the specificity by comparison (see section Quality assessment of Svhip-trained classifiers). All three classifiers show a high degree of differentiation between true positive and true negative instances, Table 1. Generated ROC-curves further support this impression as all three classifiers cover an area under the curve of 0.97, see Table 1. However, the ROC metric compares only relative label probabilities assigned to the test instances, and it should be understood as supplementary to the raw assigned labels, see Supplementary Table S2. In terms of false positive rate, the Svhip classifier with structure filter enabled reaches the level of differentiation achieved with RNAz and surpasses the non-optimized classifiers by a notable margin, see Table 1 and Figure 8.

**Table 1.** Evaluation of classifier performance using two Svhip-generated classifiers and RNAz as gold standard. We calculated sensitivity, specificity, false positive rates and raw accuracy directly from the classification results. Note that these metrics refer to both control sets combined. In any case, the resulting numbers are very similar. As an immediate observation, the data preprocessing step of filtering secondary structure alignments for significance implemented by Svhip notably causes a trade-off between sensitivity and specificity towards a lower false positive rate

Classifier	Sensitivity	Specificity	FPR	Precision	Accuracy	AUC
Svhip model	0.95	0.96	0.04	0.92	0.96	0.97
Svhip model (structure filter)	0.92	0.97	0.03	0.95	0.96	0.97
RNAz	0.93	0.97	0.03	0.93	0.96	0.97

Based on the false positive rate on both control sets, the optimized Svhip classifier shows specificity well within the lines of expectation. We note that the classifiers with no structure filter fall short in terms of specificity when compared to the other classifiers and that the higher sensitivity can thus be partially attributed to this. This as well is to be expected as we suggest a strong link between the selection of statistically significant training examples and overall performance. We note however that, in the context of genome wide screens, a low FPR is in many cases more valuable than a high sensitivity. This is the case for two reasons: Firstly, using a sliding window approach, the average screened genome will be separated into millions of overlapping windows, leading to an expected false positive classification of thousands of windows. Considering this, even a reduction of FPR by one percent can be considered beneficial, as the experimental validation of each individual discovered locus is substantially more costly than the genome screen itself. Secondly, as most genomic loci are larger than one window of around 120 nt, there is a reasonable chance for at least strongly conserved subsequences of the full locus to be classified correctly, such that many candidates can be identified even at lower sensitivity. For these reasons, we strongly suggest an optimization towards the lowest possible FPR feasible for a given use case. Aside from these considerations, we also note again that the analyzed classifiers are not optimized in terms of initial input data and serve more to showcase the overall capabilities of the pipeline. Alternative avenues of investigation here include for example the specific selection of only certain types of non-coding RNAs as input, or a restriction to certain species of origin. We further note that the stagnation of raw accuracy at around 0.96 seen here indicates that a certain threshold is probably reached in terms of classifying this particular data set and any further attempts at improvement risk overfitting.

### Analysis of two-way classification performance on Drosophila genome screen

Given the above observations, we decided to use only the classifier trained with structure filter for a non-coding RNA screen on the genome of *D. melanogaster*. A running time analysis for an increasing number of windows shows that the Svhip classifier out-performs RNAz, see Supplementary Figure S6. We expected that the overall classification accuracy of structurally conserved non-coding RNA elements would further increase when considering protein-coding sequences as a third class. This approach, however, cannot be compared to RNAz directly, which exclusively screens for conserved RNA structures in an otherwise ambiguous genomic background and does not take protein coding sequences as a separate class into account. This latter fact makes a second comparison necessary, with RNAz on the one side, the better-performing, Svhip-generated classifier on the other, and a third classifier that differentiates non-coding RNA, protein-coding and background genetic locations, which will be discussed in the following section.

We performed genome-wide screens based on Multiz alignments with *D. melanogaster* as reference and the current annotation from FlyBase v2 as ground truth (37,38). Comparison of *D. melanogaster* non-coding RNA annotations in FlyBase and RNACentral (48), a comprehensive collection of about 50 data sources, revealed a large consensus for all analyzed classes, see Supplementary Figure S10. Therefore, the well maintained data of FlyBase is used throughout this contribution. When comparing total genes covered by preprocessed alignment windows with those recovered by individual classifiers, we note that the Svhip classifier (described in the previous section) performs highly similar to the RNAz classifier in recovering snoRNAs, miRNAs and tRNAs, Table 2. This demonstrates that the Svhip pipeline is well capable of isolating high-quality training instances even from suboptimal input data. It has to be noted, however, that it is difficult to correctly determine the false positive rate of the classifiers, especially for the scenario of a full genome screen. This is primarily caused by the lack of gold standard data that would guarantee correctness of the used annotation, and the higher abundance of spurious alignments containing non-homologous or very dissimilar sequences thus introducing the possibility for reduced conservation signals despite there being a genuine conserved transcript encoded. The ambiguity and likely incompleteness of available annotations is our primary motivation for restricting the analysis to the well-studied cases of tRNA, snoRNA and miRNA genes. Although the two primary sub-types of snoRNAs are covered in the input equally well, C/D-box snoRNAs are recalled at a substantially lower rate than H/ACA-box snoRNAs, Table 2. This is most likely caused by the differences in complexity of the characteristic secondary structures of both snoRNA sub-types, (49). C/D-box snoRNAs form large loops between the conserved sequence motifs and feature very few conserved base pairs in a terminal stem. H/ACA-box snoRNAs, in contrast, typically fold into double stem loop structures with the H-box in between and the ACA-box on the 3’end of the exterior loop. This difference in recall has also been observed in previous screens with RNAz (50,51). The detection of box C/D snoRNA genes thus cannot rely on secondary structure predictions alone, calling for specialized tools (52).

**Table 2.** Comparison of the gene loci recall using both RNAz and the Svhip classifier trained on data from the Rfam database. Annotated refers to the number of genes of each analyzed RNA class annotated in FlyBase. Input alignments can be differentiated by their preprocessing status: (i) *raw* refers to the unmodified MAF blocks and (ii) *merged* to joined alignment blocks if sufficient consensus in the number of species exists. The latter was created using the Svhipmafmerge subprogram. The next column indicates the number of annotations actually covered by sliced alignment windows with at least 50% of their total length, i.e. those that are reasonably recoverable using the prediction strategy. For both RNAz and Svhip the respective recall, i.e. the number of re-covered annotations, and the resulting sensitivity is listed. For a comparison between the overlap of true positive hits see Supplementary Figure S9

				Recall	
RNA class	Annotated	MAF format	Covered	RNAz	Svhip
tRNA	290	raw	149	118 (79.19%)	120 (80.53%)
		merged	260	207 (79.61%)	210 (80.77%)
miRNA	477	raw	208	161 (77.40%)	159 (76.44%)
		merged	367	267 (72.75%)	270 (73.57%)
H/ACA-box snoRNA	140	raw	53	40 (75.47%)	51 (96.23%)
		merged	124	83 (66.94%)	100 (80.65%)
C/D-box snoRNA	142	raw	46	7 (15.21%)	9 (19.56%)
		merged	113	28 (24.78%)	21 (18.58%)

While analyzing the Multiz whole genome alignments we noted that a primary issue in the screening for conservation signals is the quality and coverage of the input alignments provided. Even a hypothetical ideal classifier would fail to recover all known annotated genes if the total coverage of the provided MAF blocks for these genes is insufficient. To investigate the magnitude of this problem, we analyzed the number of annotated genes theoretically covered by sliced alignment windows and discovered that in most cases less than half are actually present in the final input with sufficient coverage, Table 2. In general the more species are included in the alignment process, the more fragmented the genome-wide alignment becomes, i.e. the number of alignment blocks increases. If a minimum alignment block length is a requirement, which is one of the filter criteria applied in rnazWindow.pl, this becomes a serious issue on genome wide screens. In many cases these short blocks still contain valid information and can be combined into larger blocks using a quite simple approach as implement in mafmerge of the Svhip software, see Material and Methods for details. Independent of the classifier, a substantial increase in both gene coverage and total recall of ncRNA genes is observable for the merged alignments. This indicates that such preprocessing adds substantial value to the analysis.

Analyzing all hits with a minimal cutoff of *P* > 0.5, we get 48,217 total hits with Svhip and 46,596 with RNAz, respectively. Of these, 29,015 (62%) are shared between the two, suggesting a relatively high consensus. In general, the confidence or *P*-value distribution of hits looks different for both classifiers, which is presumably an artifact of underlying model architectures, as LibSVM and scikit-learn diverged in development a substantial time ago, even though the latter’s SVM implementation is built on top of the former. Of the shared hits, 4,183 map onto existing annotations of either coding or non-coding genes, or 6,941 in the case of Svhip and 6,717 for RNAz. Considering the very similar proportions of total hits and fractions that map onto existing annotations, we come to the conclusion that the false positive rate of the Svhip classifier is likely at least as good as that of RNAz for this confidence level. Together with the observations from the previous section we conclude that our classifier is about as efficient for the purpose of *Drosophila* non-coding RNA genome annotation as the RNAz framework.

### Analysis of protein classification performance on the Drosophila genome screen

A new classifier for the differentiation of protein-coding sequences and genomic background was trained based on the data obtained from InsectBase. The entire pool of training data from the ncRNA classification experiment, i.e. both the exemplary Rfam non-coding RNA alignments and the automatically generated negative set, was reused as a negative set. The goal of this experiment was the identification of coding regions in a full genome screen under the exclusion of everything else. While generation of a three-way classifier differentiating between ncRNA, coding sequences and genomic background is in principal possible with Svhip, it showed subpar results in this particular use case when compared with the usage of two individual classifiers (data not shown).

Of the 17,835 protein-coding sequences covered by the preprocessed alignments, 13,129 were recovered by the Svhip protein classifier (recall 73.8%). Note that one gene can consist of multiple coding sequences and that such a region was counted as recovered if (≥2) consecutive hits occurred on the same strand within that gene’s boundaries. If we only count regions as recovered when at least 50% of their length is covered by hits, we still obtain a recall of 66.1%, corresponding to 11,787 recovered regions. Obviously, these numbers are heavily dependent on the quality and quantity of the available training data and thus the resulting classifier.

Since the annotation of protein-coding genes is more mature as compared to that of non-coding RNA, the estimate of the expected false positive rate (FPR) can be considered to be much more reliable in this case. Of 477,593 alignment windows not annotated as protein-coding, 54,309 (11.3%) were classified as protein-coding loci. It is of course likely that the Flybase annotation does not include all coding sequences that exist in reality, in particular taking into account that also features currently annotated as non-coding can harbor some coding potential or code for sORFS/smORFS (53). Therefore, assuming that the annotation also does not include any wrongly identified coding regions, we consider it reasonable to interpret this rate as an upper bound, that may be lowered when more accurate ground truth data becomes available.

Usually, protein-coding genes are substantially longer than the non-coding RNA genes discovered with this approach. In fact, there is not a single region in the *Drosophila* genome annotated as protein-coding that is not split over two or more consecutive alignment windows. This means that, in the case of protein-coding screens, isolated windows reported as hits are most likely false positives and can be ignored in almost all cases. Applying this correction as a post-processing step reduces the false positive rate to 34,156 (i.e. 7.2%) incorrectly classified windows.

In general, it is difficult to compare the approach outlined here with other prediction methods. Typically, a lot of contemporary tools for coding sequence prediction, like CPAT (21), CPC2 (22) and RNAsamba (54), use machine learning models on individual transcripts or genomic sequences instead of multiple sequence alignments. This also means that most of them do not take evolutionary conservation into account directly, making a fair comparison difficult due to severely differing application strategies. For a baseline estimate of reliability, we did a genome-wide screen in *D. melanogaster* using RNAcode, which also relies on alignment windows, and compared predictions with both the Svhip trained classifier and the Flybase annotation. Although the applied Svhip features are chosen for simplicity and speed 90% of all covered coding regions have been correctly identified, Supplementary Figure S7. As expected, the much more sophisticated model of RNAcode outperforms Svhip. When comparing unannotated loci of both approaches in total 77,181 have been identified. In 30% of all cases the tools agree while Svhip and RNAcode predict additional 29% and 40%, respectively. One has to consider these loci as seeds for an in depth downstream analysis that tries to extend these seeds to complete coding sequences.

### Chaining of classifiers for reduced FPR

In an attempt to further reduce the expected false positive rate of the non-coding RNA classifier, we investigated ways to combine the previously described classifiers. The goal here was to effectively remove as many coding sequences from the non-coding RNA classification pool as possible as these form the largest chunk of all annotated data. It can be shown that there is a substantial overlap between potential for structure conservation in non-coding RNA and coding RNA since the conservation of base triplets tends to indirectly conserve (possibly random) secondary structures, Supplementary Figure S8. In this interpretation, the potential for conservation of secondary structure is highest in genuine non-coding RNAs and lowest in the genomic background, while coding sequences exhibit an intermediate conservation level. Thus, their removal by a preceding classification step based on coding potential could substantially reduce the margin of errors in a subsequent, conservation-based non-coding RNA screening. In general, the non-coding RNA classifier outlined in "Analysis of two-way classification performance on Drosophila genome screen" can be assumed to have a worst-case FPR of


(7) $$\begin{equation*} \operatorname{FPR}= \frac{\operatorname{FP}}{\operatorname{FP}+ \operatorname{TN}} = \frac{12,542}{928,728} =1.4\% \end{equation*}$$

or, in the case of merged alignments,


(8) $$\begin{equation*} \operatorname{FPR}= \frac{\operatorname{FP}}{\operatorname{FP}+ \operatorname{TN}} = \frac{50,726}{2,646,736} =1.9\% \end{equation*}$$

where FP (false positives) refers to non-coding RNA classification hits on windows not overlapping with a non-coding RNA annotation and TN (true negatives) refers to hits where coding sequence or genomic background windows are correctly identified as not being non-coding RNA. We explicitly counted here all potentially incorrect hits—and not only the high-confidence hits—as we assume that the probability of overlapping with genuine but not annotated genes is higher. If the goal is to estimate an upper bound on the FPR given the classifier at hand, then all hits irrespective of assigned probability should be counted.

There are two obvious options to attempt an exclusion of coding sequences from the false positive set during classification. One is to first make a full genome screen using the protein model from "Analysis of protein classification performance on the Drosophila genome screen", and subsequently classify the remaining alignment windows. In principal this introduces the possibility of misclassifying non-coding RNAs as coding, which then subsequently disappear from the pool of true positive non-coding RNA hits. During our analysis, however, this effect appeared to have no relevant impact on non-coding RNA classification. Unfortunately, this approach resulted only in a marginal reduction of FPR, which fell to 1.3% due to 206 alignment windows being removed from the false positive set.

The second option involves retraining the classifier after also including the protein-coding training set as part of the negative set for non-coding RNA classification, thus attempting to generate a classifier that more directly excludes coding sequences instead of only being based on the original negative set of synthetic alignments. Marking all training examples in the protein classifier training set as background (relative to the class of non-coding RNA) and retraining the non-coding RNA classifier with Svhip using the same parameters, yields a drastically improved FPR on the annotated *Drosophila* genome:


(9) $$\begin{equation*} \operatorname{FPR}= \frac{5,290}{928,728} = 0.6\% \end{equation*}$$

It should be noted that this classifier, as outlined in the result section "Analysis of two-way classification performance on alignments of known ncRNAs", only uses the features of SCI, *z*-score of MFE, and entropy, as it was noted that the more coding-sequence specific features do not substantially add to the non-coding RNA classification performance. These features were thus excluded from the training set for this experiment.

We conclude that, depending on the use case, it may be worthwhile to manually add known negative examples to the training set instead of relying exclusively on the artificial generation of negative training instances.

### Performance of a three-way classifier

The Svhip pipeline allows for the generation of a three-way classification model. We compared the efficiency with the chaining of multiple classifiers for different purposes as described in the previous section. For this experiment, the same training data was used as for training of individual classifiers. We note, however, that the three-way classifier performs notably worse using the same training data in a direct comparison. For instance, the number of correctly recalled protein-coding regions drops from 13,129 to 9,798 using this approach and the FPR becomes 5%. Due to these notable drawbacks, we limit the more in-depth analysis to the previously presented approach. On the other hand, preliminary attempts on yet incomplete training data yielded promising results for some more specific applications. We are therefore convinced that this approach may be a viable tool to consider for certain use cases. A more detailed analysis, however, is beyond the scope of this initial report.

## DISCUSSION

The Svhip software was used in different classification scenarios to illustrate the applicability with different research goals in mind. It can serve as both a tool for a quick screen of potential coding regions as well as for an in-depth analysis of non-coding RNAs across a variety of species. Although it was not the main motivation for implementing Svhip, we found that its runtime even for a rather small set of 2,000 alignment windows is already about 20% shorter compared to RNAz. A trend that is expected to get even more pronounced for genome-wide screens. We hypothesize that this speedup is a result of the efficient implementation of the SVM approach in scikit-learn in comparison to the old libSVM version used when RNAz was trained in 2010. It can be demonstrated that Svhip is also applicable in a more specialized scenario such as the search for high-confidence hits for coding potential in genes already assumed to encode non-coding RNAs with different biological functions. To our knowledge, no other tool currently unites these functionalities and such an investigation would typically require to make a screen with one specialized tool and another, already established annotation in mind. This use case has substantial downstream potential, as there is evidence that non-coding RNAs containing protein-coding subsequences are yet not close to being fully investigated (55,56).

Our results clearly show that Svhip generates accurate classifiers with high recall and moderately low false positive rates even from subpar training data. The set is based on the first 50 Rfam alignments manually selected for known and significant secondary structure, Supplementary Table S1. Depending on the application much more care could be taken in order to assemble an appropriate training set from available, e.g. domain of life or clade specific, data sources. We used the Rfam database as reference mainly because RNAz was trained on Rfam alignments and for the non-coding RNA application we intended to reproduce its implementation as proof of concept. The situation is similar for the training data of the protein-coding classifier and even more obvious for dual functional RNAs, i.e. those that act as non-coding as well as protein-coding RNA. With Svhip we provide a standardized framework enabling users to exploit current data and the most recent available knowledge in order to approximate a ground truth or gold standard as good as possible. The tool is primarily intended for genome-wide screens instead of the classification of single alignments of full-length sequences, which makes the classification task already more difficult at the outset. In cross-validation scenarios using subdivisions of our training data, we obtain recalls and false positive rates that are markedly superior to the data obtained from a genome screen, as the results sections Analysis of two-way classification performance on alignments of known ncRNAs and Analysis of two-way classification performance on Drosophila genome screen indicate. One problem here is the slicing of genomic regions into overlapping windows, a process that inherently destroys certain conservation and structure signals at the window boundaries. An important, yet insufficiently addressed problem is the classification of long non-coding RNAs in genome-wide screens. Long non-coding RNAs are in many ways more difficult to classify by window-based approaches because focusing on local features like secondary structure conservation becomes less viable for longer candidate sequences. Approaching this problem will be a goal of future investigation. Potential solutions include better methods to join related, but not directly adjacent hits within the same gene and improving training set composition for this specialized problem.

The software could be improved further by incorporating specifically designed clustering approaches for given groups of hits. As noted above, in many cases it may be insufficient to simply group adjacent hits together and declare them to be part of the same gene or region. This simplified approach is problematic in particular if either a very long coding sequence or a long non-coding RNA is to be analyzed. In the former case, the large number of successive alignment windows involved naturally increases the chance for false-negative hits, and any false negative would lead to an interruption of the chain and thus to a misclassification as two protein-coding regions instead of one. In the latter case, structure conservation or the overall presence of stable secondary structure is usually not evenly distributed, again leading to accidental subdivision of loci. Both of these problems have been observed in our case studies. The situation is complicated further by the presence of introns and alternative splicing events. These shortcomings emphasize the necessity to devise better methods for identifying clusters of hits, which will be the subject of future work.

## CONCLUSION

The software Svhip serves as a framework to train, test and apply machine learning classifiers in full genome screens for the annotation of protein-coding or non-coding RNA genes. We demonstrated the usage of the pipeline in a number of different scenarios and obtained comparable classification results to the RNAz framework when investigating non-coding RNA classification. The primary advantage of Svhip is the ability to in- or exclude different data sources on the fly, and to fine-tune parameters for different use cases. Further work is required to evaluate the applicability in different scenarios. One prime target is an in-depth investigation of plant genomes, many of which are currently lacking accurate and complete annotations. A particular challenge there is the high presence of gene duplication and transfer events, increasing the difficulty of the data preprocessing, and a highly volatile alignment quality and structure. We envision that the adaptability of the pipeline will serve to further improve the annotation of many different species.

## Supplementary Material

lqad072_Supplemental_File

## Data Availability

Training data, models and data used in generation of figures can be freely accessed on Zenodo (doi: 10.5281/zenodo.8120827). Additionally, a UCSC track hub provides access to the underlying data of the Svhip and RNAz comparison on a *D. melanogaster* genome-wide screen. See https://www.bioinf.uni-leipzig.de/publications/supplements/22-003 for details. The Svhip source code is additionally uploaded to Zenodo (doi: 10.5281/zenodo.8119561).
