# Supplementary material for: Tailored machine learning models for functional RNA detection in genome-wide screens
Source: NAR Genom Bioinform. 2023 Aug 21;5(3):lqad072. doi: 10.1093/nargab/lqad072 (PMC10440787; doi:10.1093/nargab/lqad072)
Supplement: lqad072_Supplemental_File [file lqad072_supplemental_file.pdf]

Supplemental Material for
Tailored machine learning models for functional RNA detection in genome-wide screens

Christopher Klapproth[1,2], Siegfried Zöztsche[1], Felix Kühnl[1], Jörg Fallmann[1], Peter F. Stadler[1,3,4,5,6], and Sven Findeiß[1,*]

[1] *Leipzig University, Bioinformatics Group, Department of Computer Science and Interdisciplinary Center of Bioinformatics, Härtelstrasse 16-18, D-04107 Leipzig, Germany,* [2] *ScaDS.AI Leipzig (Center for Scalable Data Analytics and Artificial Intelligence), Humboldtstraße 25, D-04105 Leipzig, Germany,* [3] *Max Planck Institute for Mathematics in the Science, Inselstraße 22, D-04103 Leipzig, Germany,* [4] *University of Vienna, Institute for Theoretical Chemistry, Währingerstraße 17, A-1090 Vienna, Austria,* [5] *Santa Fe Institute, 1399 Hyde Park Rd., Santa Fe NM 97501, USA, and* [6] *Universidad Nacional de Colombia, Facultad de Ciencias, Bogotá, D.C., Colombia*

[*] *corresponding author: sven@bioinf.uni-leipzig.de*

Table S1: Selected non-coding RNAs for usage in classifier training as obtained from the Rfam database. We only selected non-coding RNA families that (1) show sufficient evidence of structure conservation, and (2) comprise at least 50 sequences, either in the seed alignment, or otherwise in the regular alignment. Note that the number of training instances varies with the initial number of sequences, length of alignment and number of windows reduced during filtering. RNA families that did not yield any training instances after all filtering steps were excluded from this list.

ncRNA	number of training instances
RF00028: Group1CatalyticIntron	37
RF00079: OmrAB	110
RF00230: Tbox	10
RF00052: lin-4 miRNA	110
RF00029: Group2CatalyticIntron	39
RF00155: SNORA66	43
RF00091: SNORA62	109
RF00045: SnoRNA73	270
RF00103: miRNA1precursor	108
RF00114: ribosomalS15leader	97
RF00026: U6 Spliceosomal RNA	32
RF00131: miRNA30precursor	110
RF00089: SNORD31	19
RF00015: U4 Spliceosomal RNA	102
RF00051: miRNA17precursor	110
RF00153: SNORD62	53
RF00020: U5 Spliceosomal RNA	160
RF00073: miRNA156precursor	176
RF00056: SNORA71	66
RF00002: 8SrRNA	57
RF00047: miRNA2precursor	107
RF00106: RNAI	216
RF00090: SNORA74	210
RF00066: U7snRNA	64
RF00144: miRNA199precursor	110
RF00007: U12 Spliceosomal RNA	256
RF00030: RNaseMRP	187
RF00074: miRNA29precursor	109
RF00178: miRNA24precursor	110
RF00012: U3SnoRNA	299
RF00229: IRES	224
RF00009: RNaseP	322
RF00174: Cobalaminswitch	238
RF00129: miRNA103precursor	110
RF00003: U1Spliceosomal	170
RF00008: Hammerhead Ribozyme	98
RF00004: U2Spliceosomal	271
RF00076: miRNA181precursor	110
RF00138: SNORD16	34
RF00092: SNORA63	130
RF00005: tRNA	106
RF00019: YRNA	187
RF00150: SNORD42	1
RF00001: 5SrRNA	152

Table S2: Non-coding RNA classification results comparing performance of two `Svhip`-generated classifiers using `RNAz` as a gold standard. The test set used was originally utilized in the evaluation of the `RNAz` software [31] and contains both well-established structured non-coding RNA alignments as well as alignments taken from random genomic locations as a control (Randomized control in the table). We extended the control set with a set of alignments simulated using `SISSIz` to account for the fact that simulated alignments were used during training set generation (`SISSIz` control in the table). As `SISSIz` produces substantially less reliable results on pairwise alignments, these were discarded from the data set. Class labels assigned were either *RNA* for supposed alignments with secondary structure conservation, or *other* for everything else.

classifier	test set	RNA	other	correct label [%]
`Svhip` model	ncRNA	2896	164	94.64
	`SISSIz` control	102	2958	96.67
	Randomized control	142	2918	95.36
`Svhip` model (structure filter)	ncRNA	2819	241	92.12
	`SISSIz` control	85	2975	97.22
	Randomized control	78	2982	97.45
`RNAz`	ncRNA	2859	201	93.43
	`SISSIz` control	67	2993	97.81
	Randomized control	120	2940	96.08

Table S3: Randomly selected proteins from the *D. melanogaster* annotation used in training of the coding sequence classifier. The list below contains all those that were processed using the `Svhip` pipeline that produced feature vectors used in training.

protein name	# training instances	# sequences
AttC	174	97
Fgop2	129	116
Cyp6a19	248	29
Idgf5	264	116
MagR	14	116
Alg10	252	113
Bro	73	115
Ercc1	59	31
Capa	220	95
Atg3	383	41
Membrin	151	116
Crk	91	57
CapaR	278	66
mat	77	114
dgt2	66	116
Arc1	117	114
Gr85a	207	93
AQP	148	117
eRF1	247	117
Fibp	169	108
MED10	53	117
List	109	20
Hmgcl	68	13
Ing3	103	14
CBP	45	16
Drip	97	115
gammaCOP	413	53
CSN6	146	117

(a)

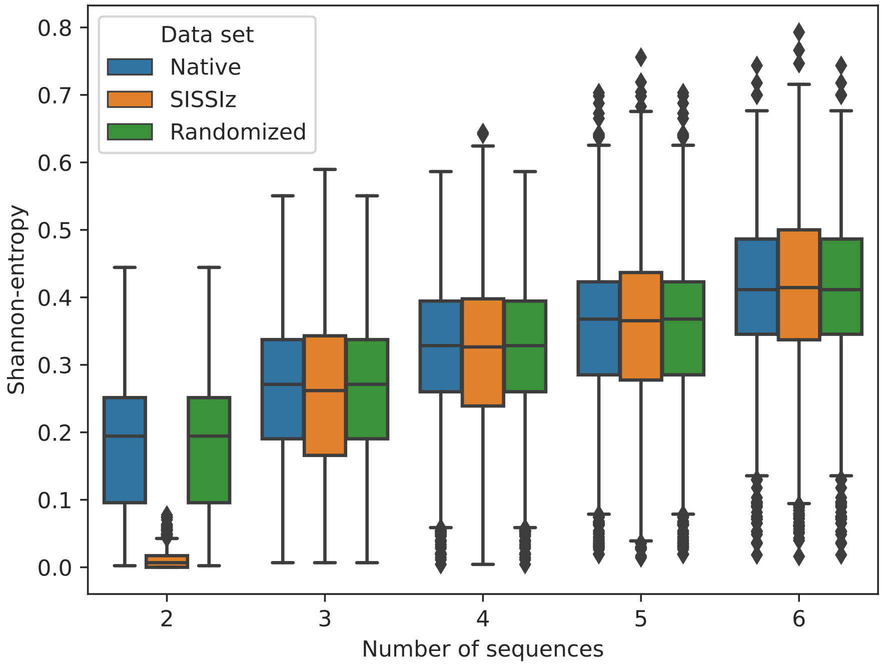

(b)

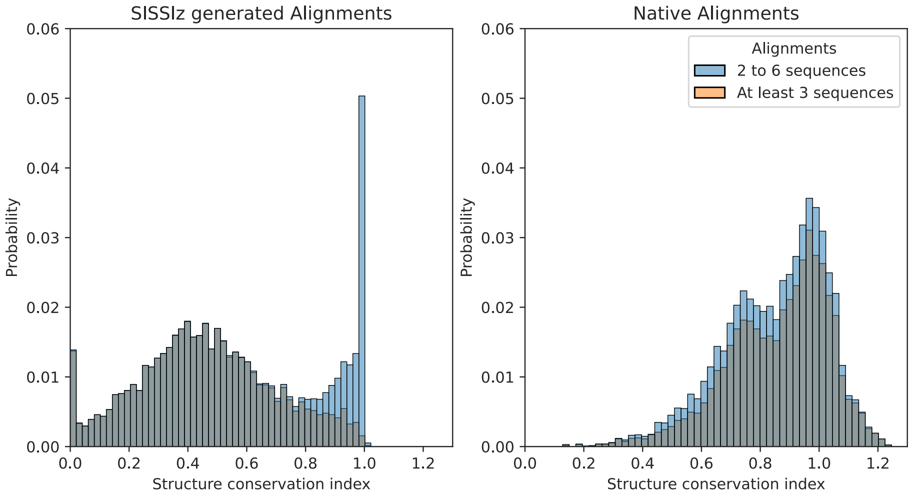

Figure S1: Limitation of `SISSIz` to simulate pairwise alignments in the context of control set generation. (a) `SISSIz` generates synthetic alignments of Shannon-entropy close to zero when provided with input alignments containing only two sequences. This implies alignments containing almost or completely identical sequences. (b) These alignments result in an artificially inflated Structural Conservation Index (SCI) of or near 1, as can be seen in the bottom left Figure. If pairwise alignments are subsequently excluded from the data set, the SCI peak at 1 disappears. This serves to eliminate unrealistic control instances from the set. The effect of removing pairwise alignments is overall moderate, bottom right Figure. Note that this issue does not occur for the more straightforward shuffling approach employed by the approach implemented in `RNAzRandomize.pl` as alignment columns are simply swapped using constraints instead of newly generated. However, the simulation approach employed by `SISSIz` still has the advantage of generating genuinely new and biologically plausible alignments via the simulation of an evolutionary tree. Therefore, alignments containing only two sequences are removed from the corresponding test set.

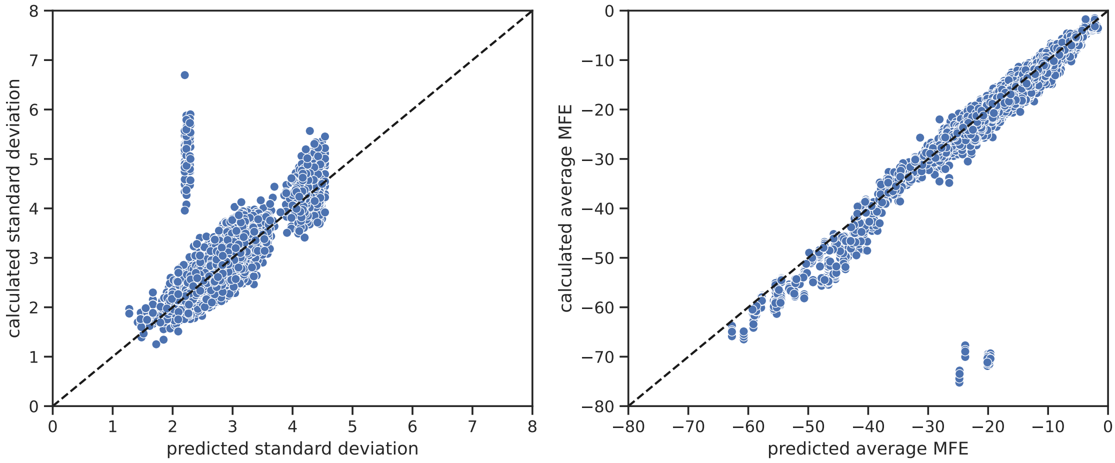

Figure S2: Individual predictions of average MFE and standard deviation of our SVR model. Values are used for the prediction of the alignment-wide mean z-score of MFE. Data points correspond to the predicted values in main Figure 4.

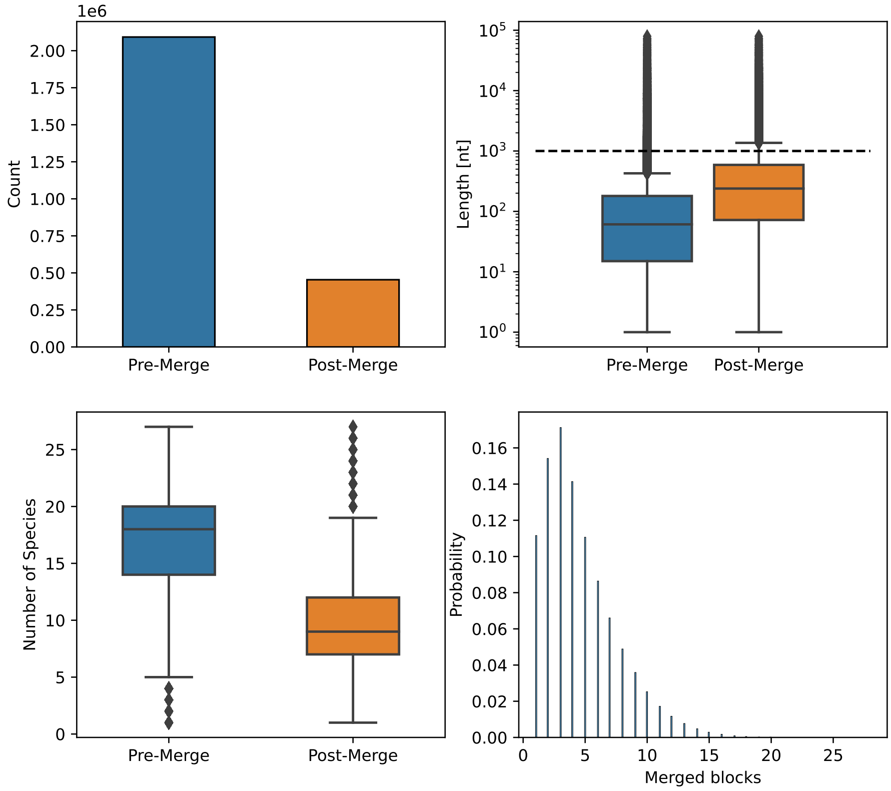

Figure S3: Statistics of merged MAF alignment blocks in the *D. melanogaster* whole genome alignment with 27 insect species. Blocks were merged up to a maximum alignment length of 1,000 nucleotides. Upper left: Total number of individual MAF alignment blocks before and after the merge step. Upper right: Distribution of lengths of blocks in nucleotides with the length cutoff as a dashed line. Lower left: Number of species per aligned block. Note that at a later processing step the number of aligned sequences is cut to a maximum of six by the `rnazWindows.pl` tool. Lower right: The probability of a given number of blocks being combined into a larger block. On average between two and four blocks were merged. Note that a number of one block here refers to a given block just being retained as it is and not combined with any others.

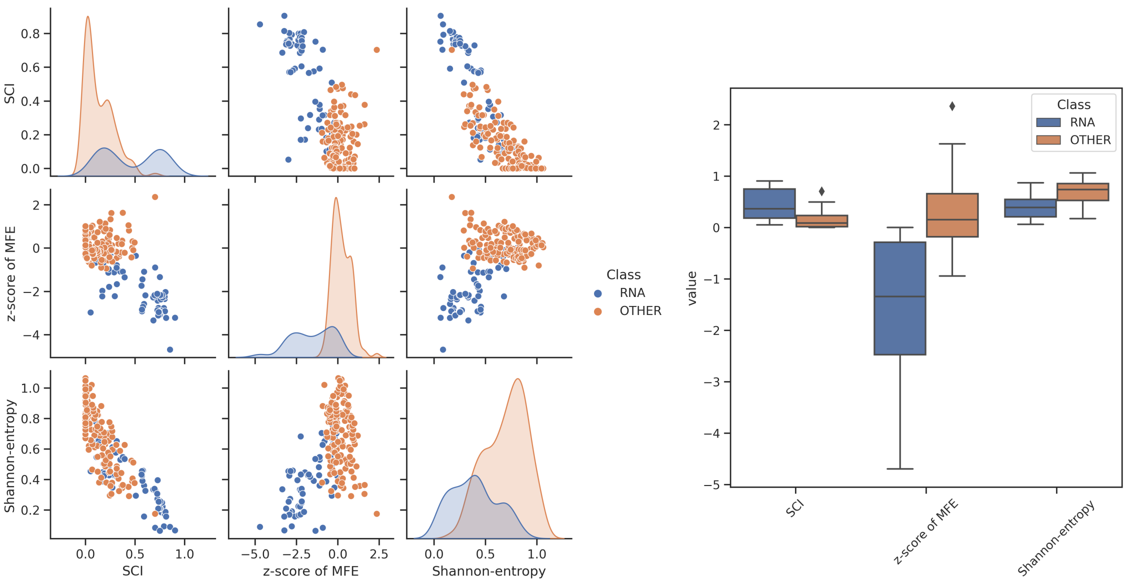

Figure S4: Graphical output automatically generated by `Svhip` for the alignment of bacterial RNAse P (Rfam ID: RF00010) RNA sequences. For an explanation of the output, refer to Figure 6. It can be assumed that the bimodal form of the RNA distribution and the strong partial overlap with the control group is partially caused by the presence of both prokaryotic and eukaryotic sequences in the initial input file, resulting in suboptimal alignments for a non-negligible number of alignment windows. In contrast to only the bacterial RNAse P alignment (RF00009), the class separation is less obvious in this particular case. In general, this figure serves to illustrate that using suboptimal alignment data as initial input (i. e. alignments of genetically very different species) may result in the selection of suboptimal alignment windows and thus potentially in less clear class separation.

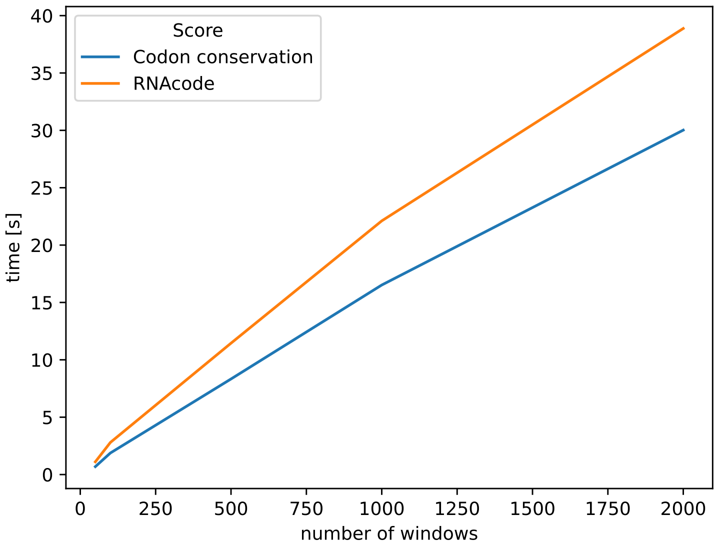

Figure S5: Comparison of the run time between `RNAcode` using only the best hit per alignment and the calculation of the codon conservation score. The first 2000 alignment windows were extracted from the Y chromosome MAF alignment of the *Drosophila* genome, and run time was measured on a continuous basis. The trend of the CCS outperforming the `RNAcode` score in the long run due to its lower computational complexity is clearly visible.

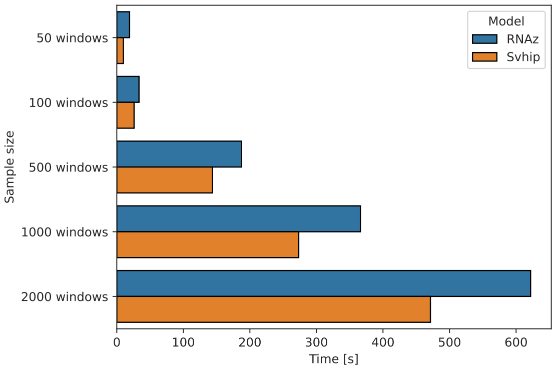

Figure S6: Run time comparison of `Svhip` and `RNAz` on an increasing number of windows sampled from chromosome Y.

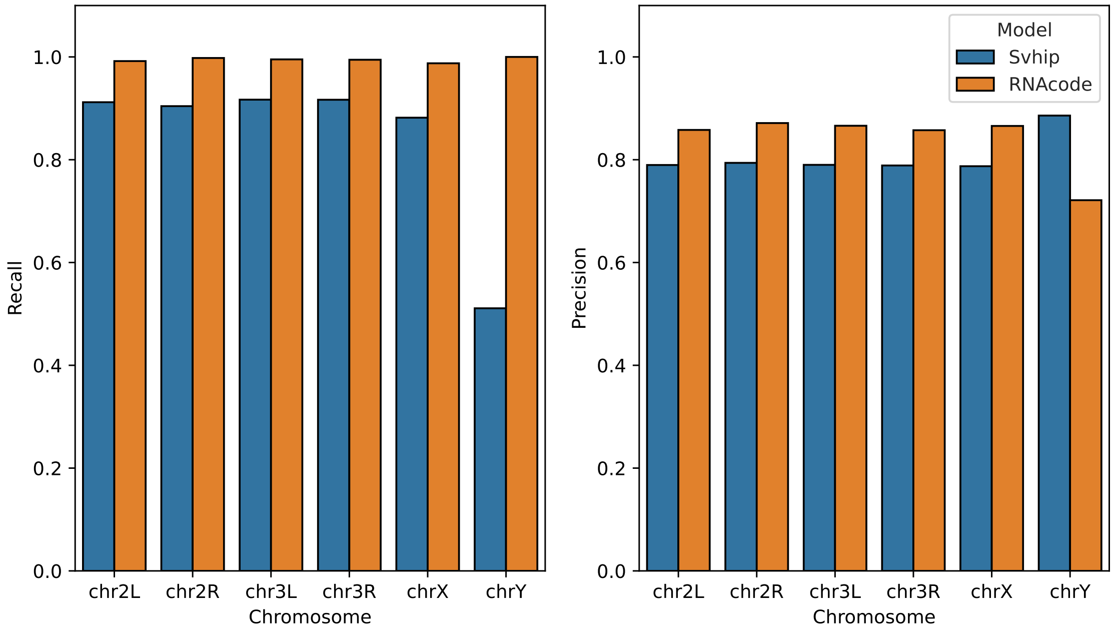

Figure S7: Comparison of predicted coding regions between the `Svhip` trained classifier and `RNAcode`. Overall recall rates of known coding regions in the FlyBase annotation v.6.46 split by the chromosomes of *Drosophila melanogaster*. Note that compared to the analysis in the main text, two modifications had to be made for a balanced comparison: First, only alignment windows with at least three sequences were considered, as `RNAcode` requires this number as a minimum. Second, we reduced the test set to alignment windows that are either completely annotated as coding region (positive set) or have no overlap at all (negative set). This was done for a fair comparison as both tools handle 'loose ends' of sequences differently (`RNAcode` provides nucleotide-exact predictions, while `Svhip` makes a binary classification for the complete window). Chromosome Y is substantially shorter in sequence than the others, thereby being difficult to compare in relative numbers.

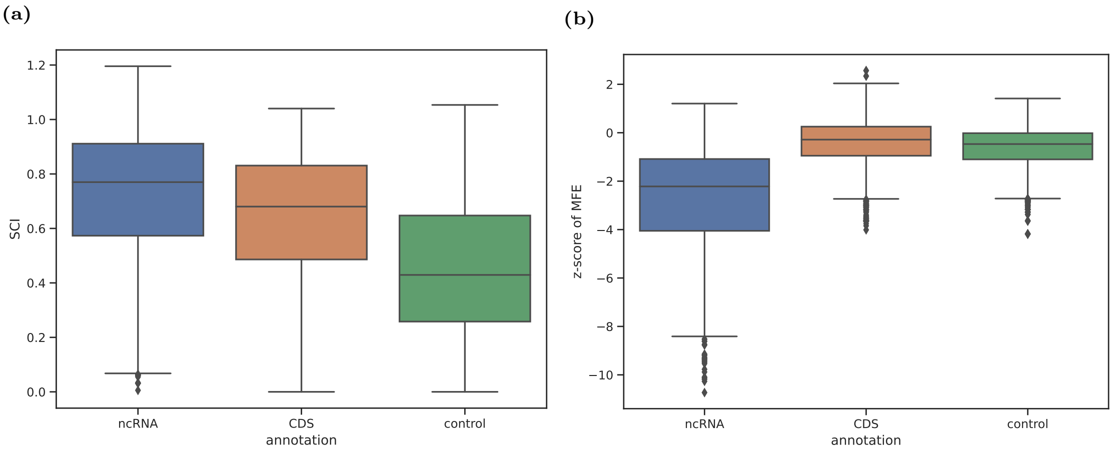

Figure S8: Distribution of secondary structure conservation features in protein-coding sequences when compared with non-coding RNA and an ambiguous background. The training data for the generation of the protein coding SVM model as described in Section Materials and Methods - Test data preparation was used. (a) Substantial differences between the SCI in coding sequences and the control can be observed. (b) This does not hold for the z-score of MFE, which yields very similar values.

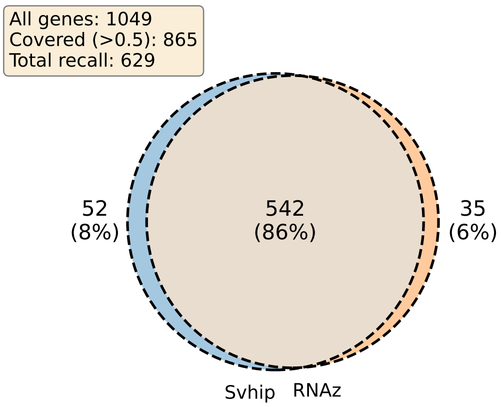

Figure S9: Venn diagram comparing true positive hits shared between the `Svhip` and `RNAz` classifier. True hits for miRNAs, tRNAs and snoRNAs were summed up. In total, `Svhip` correctly identified 52 loci that `RNAz` missed, while `RNAz` found 35 loci not found with `Svhip`. 629 hits are compared in total.

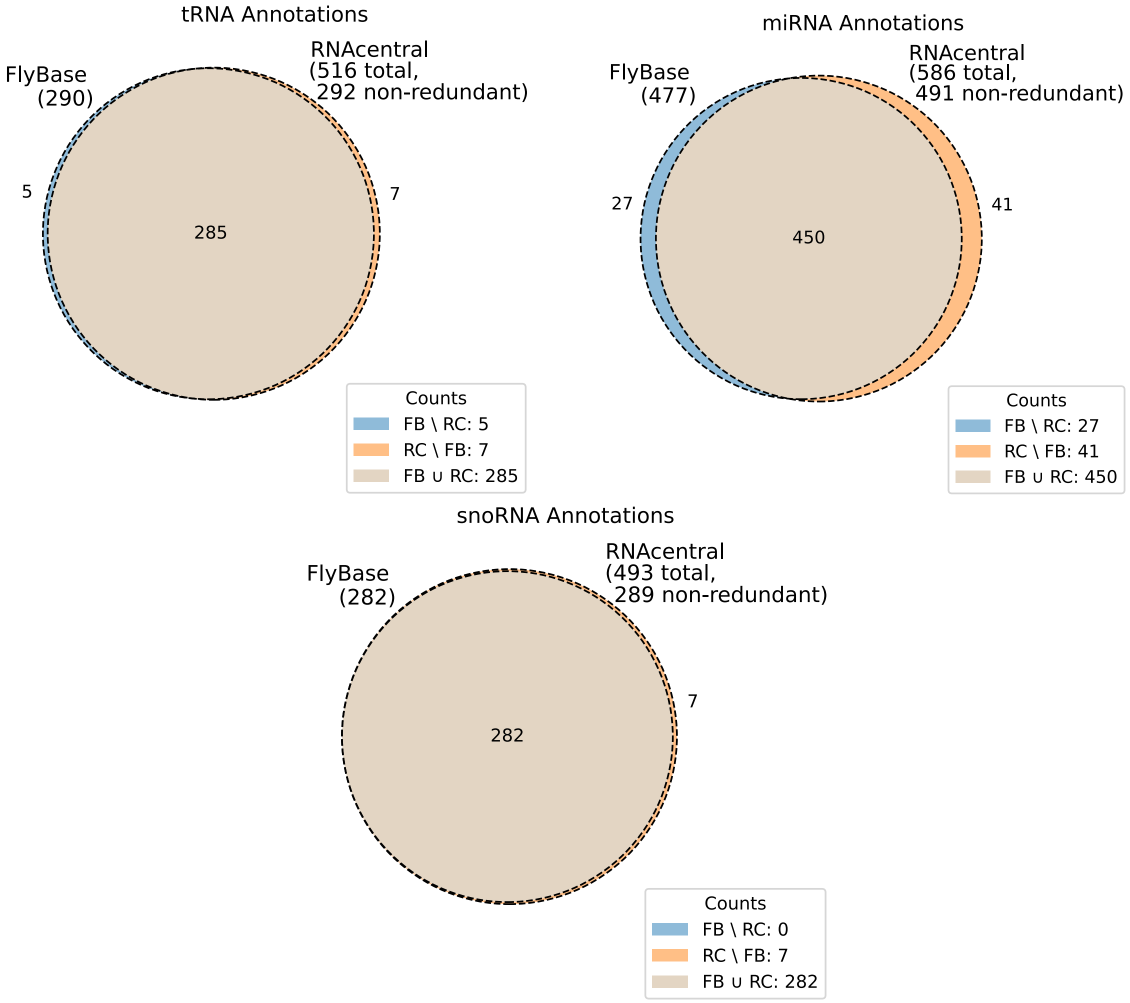

Figure S10: Comparison of non-coding RNA class annotations in `FlyBase` and `RNAcentral`. The Venn Diagrams show for all three classes a nearly complete overlap of annotated genes. Note, that `RNAcentral` contains overlapping annotations from different sources but corresponding to the same genomic locus. Therefore, counts in the Venn Diagram do not necessarily add up to the total annotation counts given in brackets directly below each database name. We therefore added the non-redundant gene counts as well for clarity. Only a very small number of non-redundant loci (7 for tRNA and snoRNA each, 41 for miRNA) not covered in the `FlyBase` remain. More than 60% of the miRNA loci (26 of 41) are located in regions annotated as repeats and therefore presumably not contained in `FlyBase`. `FlyBase` contains a comparable number of loci (5 for tRNA, 27 for miRNA and none for snoRNAs) for each RNA class that seem not to be present in `RNAcentral`. This implies that even with the additional data provided by `RNAcentral`, the calculated recall rates (Table 2 in the main text) would differ only marginally if at all.
